# Obesity-related alterations of intrinsic functional architecture: a resting-state fMRI study based on the human connectome project

**DOI:** 10.3389/fnut.2025.1559325

**Published:** 2025-05-29

**Authors:** Tongtong Wang, Todd Jackson, Matthew Lock, Hui Li, Jin Yan, Qian Zhuang, Shuaiyu Chen

**Affiliations:** ^1^Center for Cognition and Brain Disorders, The Affiliated Hospital of Hangzhou Normal University, Hangzhou, Zhejiang, China; ^2^Department of Psychology, University of Macau, Macau, Macao SAR, China; ^3^The First Affiliated Hospital, College of Medicine, Zhejiang University, Hangzhou, China

**Keywords:** obesity, resting-state fMRI, intrinsic connectivity contrast, fractional amplitude of low-frequency fluctuation, functional connectivity

## Abstract

**Background:**

Obesity, particularly in high-risk groups for food addiction, adversely impacts the brain’s functional characteristics. However, its underlying neurobiological and molecular mechanisms remain elusive. The current study adopted a data-driven approach to investigate obesity-associated intrinsic functional architecture and neurotransmitter receptor patterns.

**Methods:**

Resting-state fMRI data were acquired from 198 obese and 291 healthy weight individuals from the Human Connectome Project. Intrinsic connectivity contrast (ICC) and fractional amplitude of low-frequency fluctuations (fALFF) analyses were performed to identify the common altered brain regions and then seeds to whole brain functional connectivity (FC) analyses were conducted to determine obesity-related FC features. Additionally, the relationship between intrinsic functional characteristics and molecular imaging features was assessed to examine neurotransmitter-receptor distribution patterns underlying obesity.

**Results:**

Obese individuals, compared to healthy weight individuals, showed aberrant ICC and fALFF in both the right dorsolateral prefrontal cortex (DLPFC) and left insula. For the FC results, the obese group displayed increased FC between the right DLPFC and precuneus, left insula and left inferior parietal lobule, right DLPFC as well as decreased FC between right DLPFC and left precentral, left postcentral gyrus, and bilateral paracentral lobule. Additionally, the fALFF alterations in insula/temploral pole and also the rDLPFC-PCL FC partially mediated the relationship between body mass index and the executive function. Furthermore, cross-modal correlation analyses indicated that ICC and fALFF alterations were related to noradrenaline transporter and dopamine receptor distributions, respectively.

**Discussion:**

Together our findings suggested that obesity is associated with atypical neurotransmitter systems and dysfunctional architecture especially in the prefrontal cortex, insula, sensorimotor cortex, and default mode circuits. These may deepen our understanding the neurobiological basis of obesity and provide novel insights into neuroimaging-based treatment and intervention.

## 1 Introduction

Obesity is escalating to pandemic proportions globally. According to the World Health Organization (WHO), 43% of adults were overweight and 16% were obese in 2022^1^, highlighting obesity as a significant global public health issue (1). Obesity has negative associations with physical and mental health that include increased risk for hypertension, diabetes, anxiety disorders, and depression (2–4). Mounting evidence suggests that obesity often impairs cognitive functions, such as executive control and memory (5–7). Given such evidence, it is imperative to deepen our understanding of the neurobiological mechanisms underlying obesity.

Emerging research indicates that obesity is associated with deficits in inhibitory control and reward processing, which are reflected in functional disturbances in corresponding brain areas (8–10). For example, obese and overweight individuals exhibit hyper-responsivity in the reward areas and hypo-responsivity in areas associated with inhibitory control when exposed to high-calorie food images, compared to healthy weight individuals (11). Furthermore, obesity is associated with dysfunction in the interoceptive system, implying that affected individuals have impaired perceptions of intersensory signals, such as hunger and satiety, which lead to increased food consumption (12–14). Despite these findings, further exploration is needed to elucidate the obesity-related variations in intrinsic functional connectivity when the brain is at rest.

In recent years, resting-state functional MRI (rs-fMRI) has emerged as a promising tool for assessing spontaneous neural activities and is widely accepted in both research and clinical settings (15, 16). Examples of related approaches include fractional amplitude of low-frequency fluctuation (fALFF) and intrinsic connectivity contrast (ICC) (17, 18). fALFF measures the magnitude of low-frequency oscillations at the regional level (18) and has been used to investigate neural underpinnings of obesity (19). For instance, recent research indicates that obese individuals exhibited elevated fALFF in the dorsolateral prefrontal cortex (DLPFC), insula, and precuneus (20), compared to healthy weight controls, suggesting aberrant spontaneous fluctuations in these regions during cognitive control and emotion processing. Other researchers (19, 21) have observed discordant patterns of spontaneous fluctuations between obese samples. Additionally, previous studies have identified contrasting patterns of intrinsic activity in the default mode network (DMN) between obese samples (22–24). These studies underscore considerable heterogeneity in the intrinsic brain functional organization in obese samples, which may be partially due to variations in regions of interest (ROIs) and relatively small sample sizes (10).

Of note, aberrations in regional spontaneous activity related to obesity do not provide insights about relationship of regional aberrations in spontaneous neural fluctuations with divergent connectivity. Consequently, alongside fALFF, the well-established exploratory analytical method of “intrinsic connectivity contrast” (ICC) analysis is recommended to address this gap. ICC quantifies the strength of global connectivity patterns between a voxel and the rest of the brain, based on network theory (17). This index requires no prior knowledge or assumptions (25) and has been extensively used to investigate functional brain changes in psychiatric and neurocognitive disorders (26–28). Prior research has demonstrated that individuals with nicotine dependence exhibit ICC alterations in the medial frontal cortex (mPFC), DLPFC, and supramarginal gyrus (29). Additionally, ICC in the nucleus accumbens and caudate has been found to accurately discriminate between cannabis-dependent individuals and controls (30). However, ICC has not yet been employed to investigate the intrinsic functional organization in obesity. Furthermore, recent evidence suggests that employing multilevel indices from different perspectives may enhance the ability to comprehensively uncover intrinsic brain dysfunction, thereby facilitating a more sensitive identification of regional abnormalities (31, 32). As such, in this study, we combined ICC and fALFF to explore alterations in intrinsic brain functions associated with obesity, offering a more comprehensive analysis approach.

In addition, obesity is closely linked to disruptions in neurotransmitter systems, such as dopaminergic system and noradrenaline system. Dopamine plays a significant role in modulating appetitive behaviors through brain regions involved in reward processing (33–35). Similarly, the noradrenaline system, widely distributed throughout the central nervous system, plays a crucial role in energy balance (36, 37). A positron emission tomography (PET) imaging study showed that noradrenaline transporter (NAT) availability in the DLPFC and hypothalamus strongly correlates with changes in body mass index (BMI) following gastric bypass surgery in morbidly obese individuals (38). Notably, brain function, as represented by resting-state functional connectivity (RSFC), may be coupled with neurotransmitter systems (39, 40). For instance, GABA levels in the primary motor cortex were negatively associated with the connectivity strength of the resting motor network (40). Nonetheless, there is a need to elucidate the nature of the association between neurotransmitter systems and spontaneous neural activity in obese individuals.

To address these gaps in the literature, we employed a data-driven, multi-algorithm approach that combines ICC and fALFF to explore intrinsic functional architecture alterations associated with obesity, based on a large sample from the Human Connectome Project (HCP). Participants were categorized into the obesity (OB) and healthy weight (HW) groups based on BMI. Brain regions exhibiting concurrent ICC and fALFF alterations were selected as ROIs to investigate FC patterns associated with obesity. To examine relationships between spontaneous brain activity, intrinsic functional characteristics, and neurotransmitters, we assessed obesity-related functional abnormalities linked to the dopaminergic system, serotonin system, and NAT using a novel cross-modal data analysis approach. Based on previous studies (8), we hypothesized that obese individuals, compared with healthy weight individuals, would exhibit intrinsic functional alterations in brain areas related to executive control, motivational reward, and self-reference. These regional alterations were expected to correlate with dopaminergic and noradrenaline system distribution.

## 2 Methods

### 2.1 Participants

We selected 489 participants (290 women) from the publicly available HCP database.^2^ The final sample was determined by the following criteria: (a) exclusion of subjects lacking T1 or 3T_RS-fMRI scans; (b) screened according to body weight criteria, specifically individuals with a BMI ranging from 20 to 24 and a BMI greater than 30; (c) exclusion of subjects with missing or corrupted REST1_LR data; (d) removal of subjects with excessive head movement (exceeding 2.5 mm or 2.5 degrees in max head motion). The final cohort of 489 participants was divided into two groups: an obesity group (BMI > 30) and a healthy weight group (BMI of 20 to 24). For additional details of HCP inclusion and exclusion criteria, please see Van Essen et al. (41). All participants provided informed consent, and the entire protocol received approval from the Institutional Review Board at Washington University School of Medicine. Group differences in age, BMI, and handedness were assessed for significance using independent-sample *T*-tests. Differences in the distribution of gender and ethnicity between groups were examined using chi-squared tests. All analyses were done using SPSS software with a threshold of *p* < 0.05.

### 2.2 Neuroimaging

#### 2.2.1 MRI acquisition

The fMRI data were scanned at Washington University in St. Louis using a Siemens 3.0 T “Connectome Skyra” with a standard 32-channel receiver head coil. T1-weight structural images were acquired with the following parameters: Repetition Time (TR) = 2,400 ms, Echo Time (TE) = 2.14 ms, flip angle = 8°, Field-Of-View (FOV) = 224*224 mm, voxel size = 0.7 mm isotropic. Resting fMRI images were acquired using a gradient-echo-planar sequence with multiband factor 8, TR = 720 ms, TE = 33.1 ms, flip angle = 52°, FOV = 208*180 mm, Matrix = 104*90, voxel size = 2 mm isotropic.

The rs-fMRI data used in the current study was from the HCP database (that is “the REST1_LR run”), which lasted 15 min and included 1,200 frames. During the rsfMRI, participants were instructed to keep their eyes open and fixate on a projected bright crosshair on a dark background.

#### 2.2.2 Image data preprocessing

All data were preprocessed using the minimal preprocessing pipeline and the details on data preprocessing can be found in (42). Structured artifacts within the time series were removed by independent component analysis (ICA) and FIX (FMRIB’s ICA-based X-noisifier) (43–45). Additional preprocessing was conducted using the CONN connectivity toolbox (v. 21a; 46). The following preprocessing steps were applied to the data: (a) spatial smoothing with a Gaussian kernel of full width at half maximum (FWHM) of 6 mm; (b) bandpass filtering between 0.01—0.1 Hz; (c) denoising via the CompCor algorithm (46) by regressing out the filtered white matter, cerebrospinal fluid (CSF), effect of the rest, and head-motion (12 variables from “Movement_Regressors_dt.txt” and their quadratic); (d) detrending to remove linear trends. Given the ongoing debate regarding the use of GSR in resting-state fMRI preprocessing (47)—particularly it may abolish or reverse important rsFC results (48)—we did not regress out the global signal in this study.

#### 2.2.3 RS-fMRI data processing

**Figure 1:**
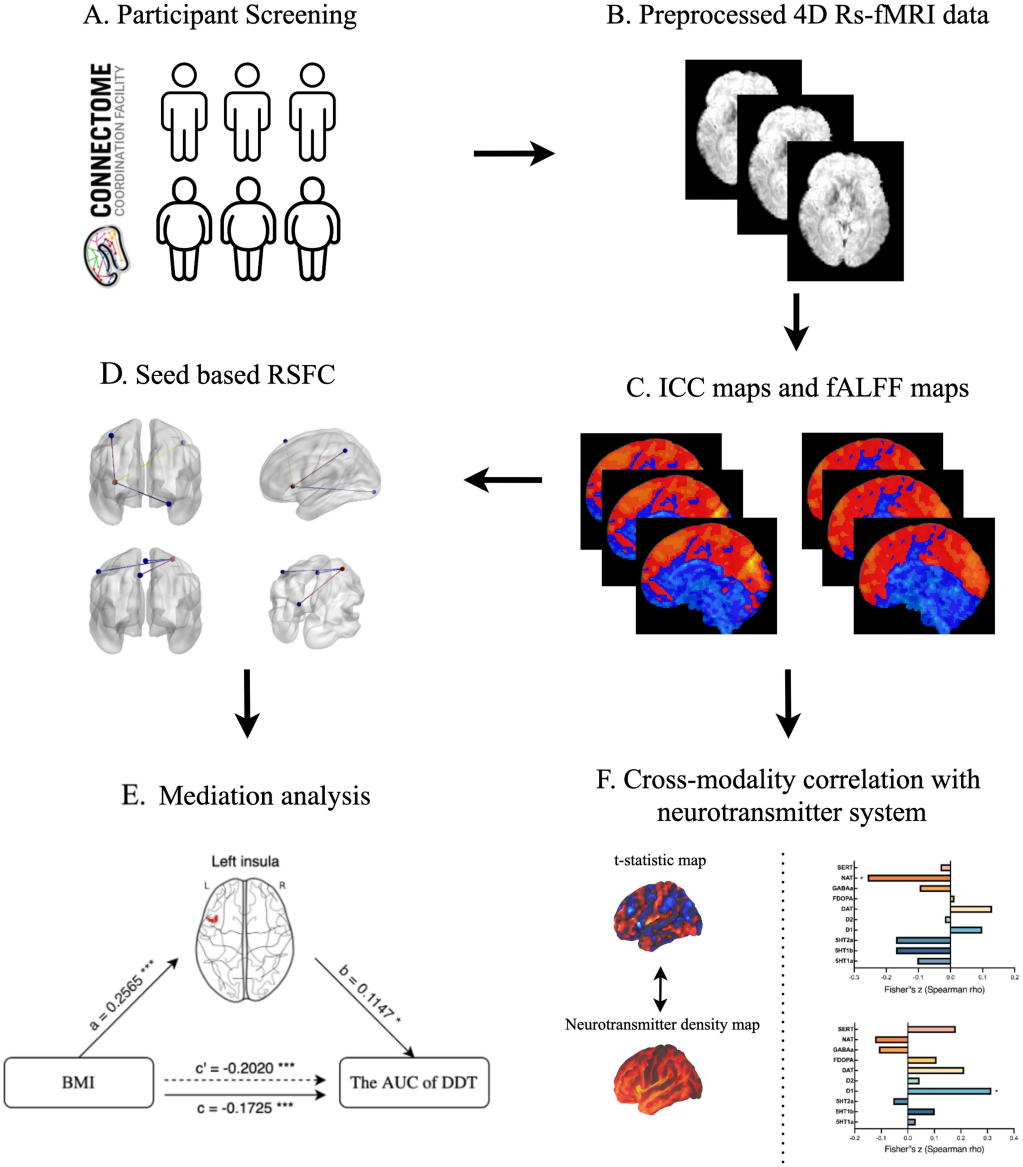
The schematic description presenting a step-by-step process of statistical analysis. **(A)** Participant selection; **(B)** Image data preprocessing; **(C)** ICC and fALFF analysis; **(D)** Seed-based RSFC analysis; **(E)** Mediation analysis; **(F)** Cross-modality correlation analysis. ICC, intrinsic connectivity contrast; fALFF, fractional amplitude of low frequency fluctuation; RSFC, resting-state functional connectivity; BMI, body mass index; AUC, area under curve; DDT, Delay Discounting Task.

The analysis flow is shown in Figure 1. All calculations were implemented using CONN software (49), with gender and age regressed as covariates.

##### 2.2.3.1 Fractional amplitude of low-frequency fluctuation

The calculation of fALFF aligns with the methods proposed by Zou et al. (18). The power spectrum was obtained by transforming the time series data for each voxel into the frequency domain using a fast Fourier transform algorithm. Subsequently, the square root of the power spectrum was calculated and the amplitude of low-frequency fluctuation (ALFF) was determined by averaging the square roots across the 0.01–0.1 Hz range for each voxel (50). Additionally, fALFF was calculated by computing the ratio of the power spectrum within the low-frequency range (0.01–0.1 Hz) to that of the entire frequency spectrum. For analyses, fALFF values were z-transformed.

##### 2.2.3.2 Intrinsic connectivity contrast

Voxel-wise global connectivity was assessed through ICC, a completely data-driven metric that does not require a preset threshold. ICC represents an estimate of the association strength between the time series of a specific voxel and all other voxels in the brain, with higher values indicating stronger connectivity. Specifically, we calculated the root mean square (RMS) of each voxel’s connections with other voxels throughout the brain based on blood oxygenation level-dependent (BOLD) time series (17). ICC values were then z-transformed for analysis.

##### 2.2.3.3 Seed-based functional connectivity (RSFC)

To further elucidate specific networks underlying observed group differences in global connectivity, a subsequent seed-to-voxel FC analysis was conducted. Seed regions were determined as spheres with a radius of 6 mm, according to the overlay brain regions of significant group difference from ICC and fALFF (17). Correlation coefficients were computed between the mean BOLD signal time series of each seed and all other brain voxels, yielding individual voxel-wise FC maps via a weighted general linear model (weighted-GLM). To normalize FC maps, correlation coefficients (r values) were transformed into Z-scores using Fisher’s r-to-z transformation.

##### 2.2.3.4 Threshold

For fALFF and ICC, obesity versus control group differences were assessed using an independent-sample *T* test with a conservative threshold of voxel-level *p* < 0.001 and cluster-level P_FWE_ < 0.05, corrected using family-wise error (FWE). To better explore obesity—related differences in intrinsic functional architecture, we applied a relatively lenient false discovery rate (FDR) correction for FC (voxel-level *p* < 0.001 and cluster-level P_FDR_ < 0.05).

### 2.3 Cross-modality correlation analysis

To investigate the relationship between receptor systems activity and spontaneous brain activity alterations, we utilized voxel-wise non-threshold T statistic maps of ICC and fALFF to assess Spearman correlations with PET-and SPECT-derived maps in JuSpace^3^ (51). Significant results were defined as *p* < 0.05 (adjusted for spatial correlation; *N* = 10,000 permutations; FDR corrected). In this study, we included 10 maps from JuSpace that represent different types of neurotransmitters for the analysis. Detailed PET and SPECT map selections are available in the Supplementary materials.

### 2.4 Mediation analysis

To further explore the association between altered brain functionality and behavioral performance in obese individuals, we utilized performance scores from executive function tasks such as the Dimensional Change Card Sort Task (DCCS, “CardSort_AgeAdj”) and Flanker Test (“Flanker_AgeAdj”), as well as impulsivity measures from the Delay Discounting Task (DDT, mean of the area under curve (AUC) variables for the $200 and the $40,000 delayed reward conditions) (52), sourced from the HCP database. With the DDT, a smaller AUC is indicative of greater impulsivity.

To elucidate the potential mediating role of the brain’s functional alterations on the relationship between BMI and behavioral data, the mediation analysis was performed with the PROCESS macro in SPSS 26.0.^4^ Similar to the prior studies (53, 54), we used the bootstrapping method (5,000 steps) to assess the significance of the mediation effect. Mediation effects were deemed statistically significant when the bootstrapped 95% confidence intervals (CI) did not encompass zero.

We conducted a power analysis using the online tool Monte Carlo Power Analysis for Indirect Effects of Mediation Models [(55), https://schoemanna.shinyapps.io/mc_power_med/] to ensure the adequacy of our sample size. The results indicated a power of 1 for a sample size of 198, assuming path correlations of 0.65 (a and b) and 0.60 (c’), with standard deviations of 1 for all paths.

## 3 Results

### 3.1 Demographic characteristics

The detailed information of the final sample is shown in Supplementary Table S1. There were no group differences in age, sex, handedness or ethnicity between the obese and healthy weight groups. There were significant differences in scores of DCCS and AUC of DDT between the two groups. Mediating effects of FC and fALFF on associations between BMI and cognitive task performance based on these measures were assessed in subsequent analyses.

### 3.2 Group differences in the intrinsic connectivity contrast

**Figure 2:**
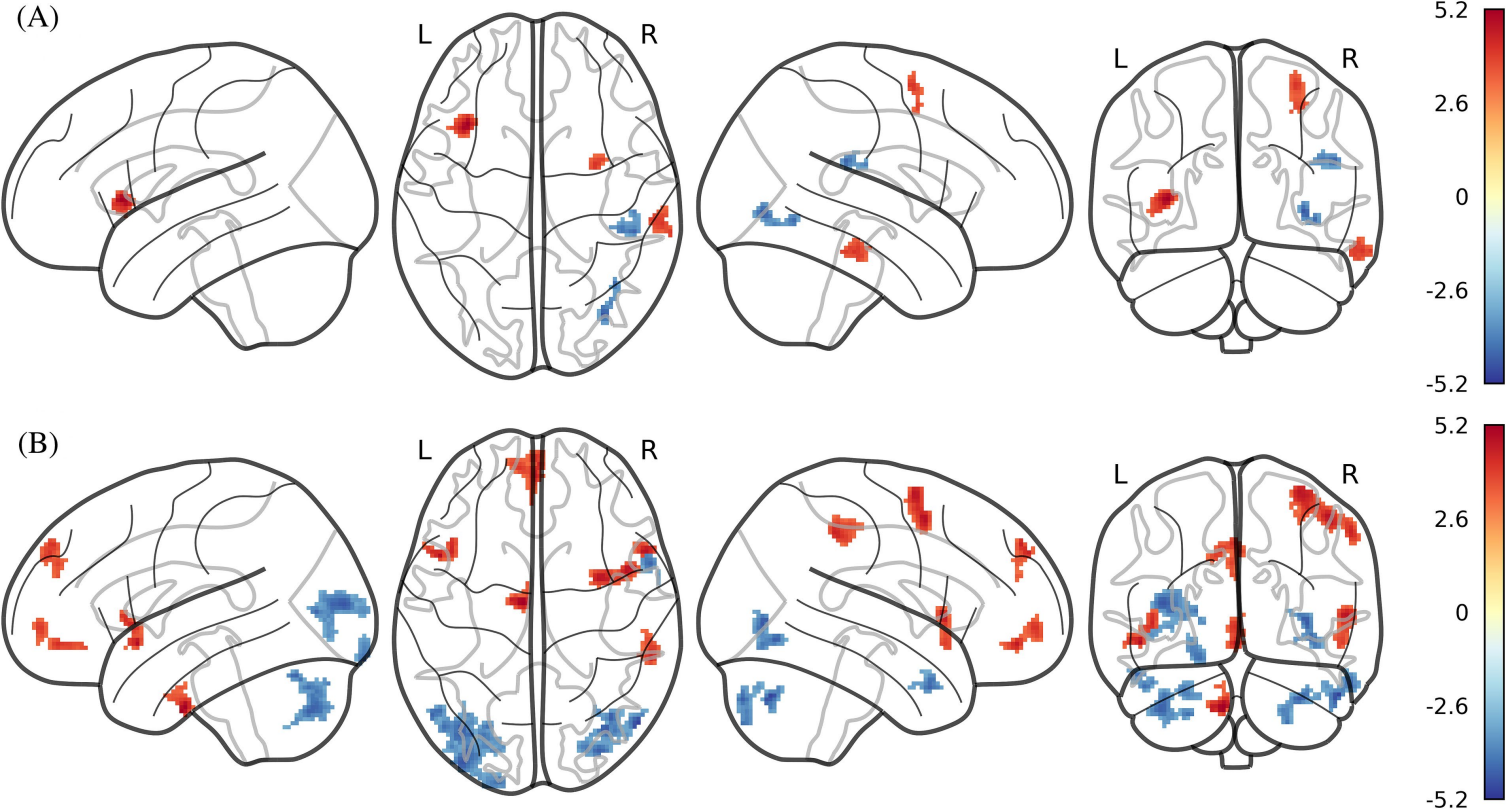
ICC and fALFF maps of statistically significant differences by two-sample *t*-test between obese individuals and healthy weight individuals (P_FWE_ < 0.05 at the cluster-level with a cluster-defining voxel-wise statistical threshold of *p* < 0.001 uncorrected). **(A)** ICC; **(B)** fALFF. ICC, intrinsic connectivity contrast; fALFF, fractional amplitude of low frequency fluctuation. Color bar indicates the T score.

Compared to the healthy weight group, the obese group exhibited significantly increased ICC in the left insula, and right DLPFC (Table 1; Figure 2A). Furthermore, brain regions with decreased ICC in the obese included the right inferior occipital gyrus (IOG) and right supramarginal gyrus compared to the healthy weight group (Table 1; Figure 2A).

**Table 1:** Significant group differences in ICC and fALFF between obese individuals and healthy weight individuals.

Index	Brain region	Cluster size (k)	Peak intensity	MNI coordinate		
				*X*	*Y*	*Z*
ICC	**OB > HW**					
	L. INS	95	5.159	−34	20	4
	R. ITG	82	4.013	64	−30	−22
	R. DLPFC	59	4.002	30	0	62
	**HW > OB**					
	R. IOG	66	−4.586	34	−76	−2
	R. SMG	73	−4.326	50	−34	24
fALFF	**OB > HW**					
	Brain-Stem	76	5.228	−6	−12	−42
	L. TPOsup	90	4.928	−48	12	−10
	L. INS		4.32	−48	18	2
	R. DLPFC	231	4.821	48	6	52
	R. FO	104	4.755	56	14	4
	L. mPFC	126	4.638	0	52	−10
	L. mPFC	131	4.465	0	54	40
	R. IPL	108	4.443	58	−40	46
	**HW > OB**					
	R. CERCRU1	62	−4.958	52	−72	−36
	L. CERCRU1	66	−4.406	−52	−68	−26
	R. CERCRU2	108	−4.501	32	−86	−38
	L. CERCRU2	245	−4.478	−38	−74	−44
	L. MOG	365	−4.627	−36	−92	10
	R. IOG	122	−4.597	38	−70	−8
	L. IOG	61	−4.242	−18	−102	−18
	R. TPOmid	55	−4.399	58	8	−32

The significance threshold was set at voxel-level *p* < 0.001 and cluster-level p_FWE_ < 0.05 for both ICC and fALFF. ICC, intrinsic connectivity contrast; fALFF, fractional amplitude of low frequency fluctuation; MNI, Montreal Neurological Institute; FWE, Family-wise error; DLPFC, dorsolateral prefrontal cortex; INS, insula; ITG, Inferior temporal gyrus; IOG, Inferior occipital gyrus; MOG, middle occipital gyrus; SMG, SupraMarginal gyrus; TPOsup, Temporal pole: superior temporal gyrus; TPOmid, Temporal pole: middle temporal gyrus; FO, Frontal operculum; mPFC, medial prefrontal cortex; IPL, Inferior parietal gyrus; CERCRU1, Crus I of cerebellar hemisphere; CERCRU2, Crus II of cerebellar hemisphere; OB, obese individuals; HW, healthy weight individuals; L, left; R, right.

### 3.3 Group differences in fractional amplitude of low-frequency fluctuation

The obese group showed significantly increased fALFF in the right DLPFC, left mPFC, right inferior parietal lobule (IPL), left temporal pole/insula, and brain stem compared to the healthy weight group (Table 1; Figure 2B). Furthermore, fALFF was significantly lower in the bilateral cerebellum crus (I and II), bilateral IOG, right middle occipital gyrus (MOG), and right temporal pole of the obese group versus healthy weight group (Table 1; Figure 2B).

### 3.4 Seed-based RSFC

**Figure 3:**
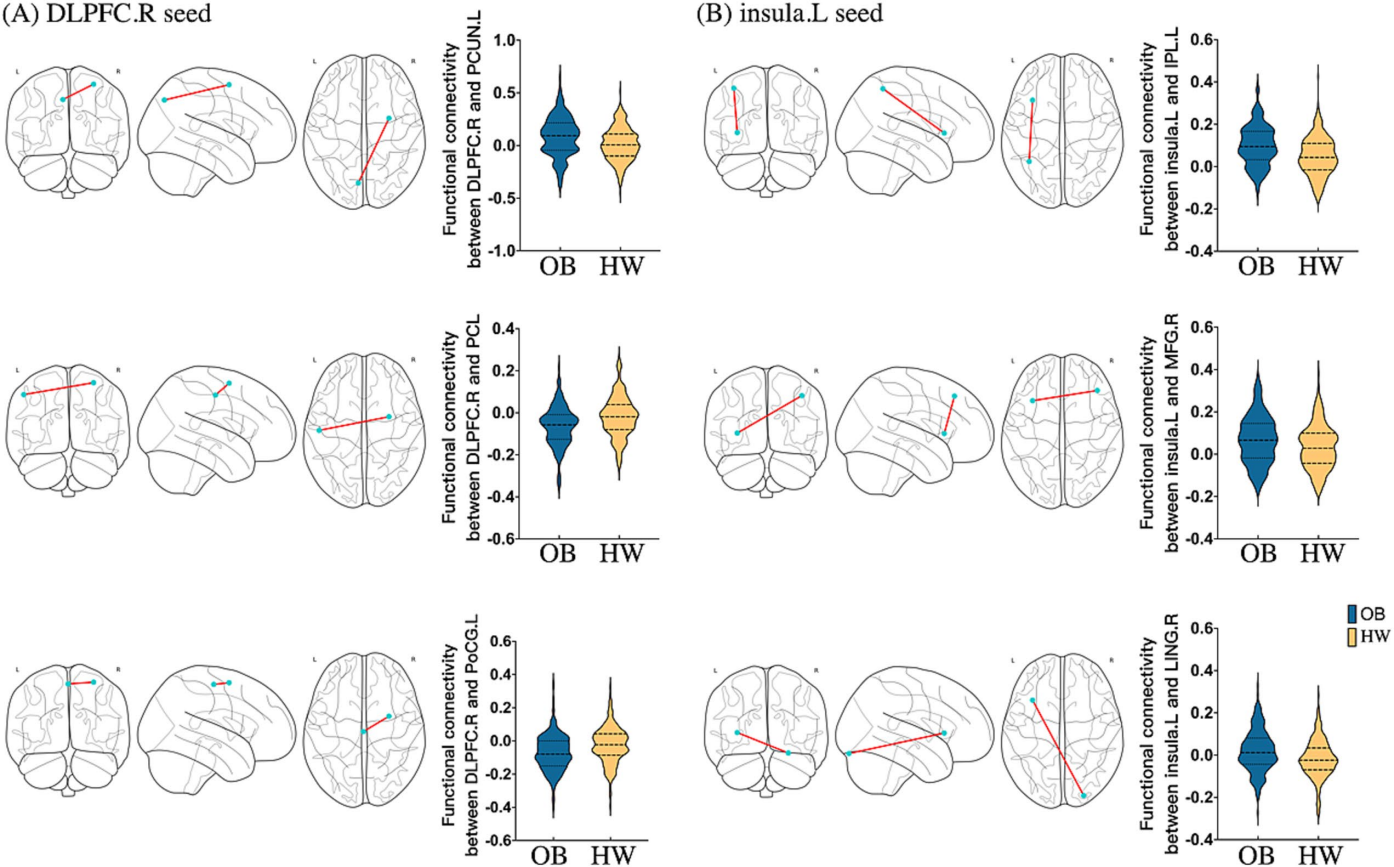
Seed-based FC maps of statistically significant differences by two-sample *t*-test between obese individuals and healthy weight individuals (P_FDR_ < 0.05 at the cluster-level). **(A)** RSFC based on the right DLPFC; **(B)** RSFC based on the left insula. DLPFC, dorsolateral prefrontal cortex; INS, insula; PCL, paracentral lobule; PoCG, postcentral gyrus; PCUN, precuneus; IPL, inferior parietal lobule; MFG, middle frontal gyrus; LING, lingual gyrus; R, right; L, left; RSFC, resting-state functional connectivity; OB, obeses individuals; HW, healthy weight individuals.

Using the right DLPFC, right IOG, and left insula as seeds, we tested differences in seed-based FC between the two groups. As for the right DLPFC seed, obese group versus healthy weight group had a stronger FC in the left precuneus (k = 194) and a lower FC in the left postcentral gyrus extending to the precentral gyrus (k = 714), as well as the bilateral paracentral lobule (PCL, k = 546) (Table 2; Figure 3). The analysis based on the left insula seed showed significantly enhanced FC in the left IPL extending to the angular gyrus (AG, k = 713), right DLPFC (k = 487), and right lingual gyrus (k = 100) for the obese group versus healthy weight group (Table 2; Figure 3). Finally, right IOG seed exhibited comparatively decreased connectivity with the occipital cortex (i.e., bilateral IOG and MOG, kR = 2,553, kL = 1943) in the obese group (Table 2).

**Table 2:** Significant group differences in seed-based functional connectivity between obese individuals and healthy weight individuals.

Seed	Brain region	Cluster size (k)	Peak intensity	MNI coordinate		
				*X*	*Y*	*Z*
R. DLPFC seed	**OB > HW**					
	L. Precuneus	184	4.842	−6	−76	44
	**HW > OB**					
	L. Postcentral gyrus	714	−4.796	−52	−16	48
	L. Precentral gyrus					
	L. Paracentral lobule	546	−4.897	0	−18	60
	R. Paracentral lobule					
L. INS seed	**OB > HW**					
	L. Inferior parietal gyrus	713	4.860	−38	−52	56
	L. Angular gyrus					
	R. DLPFC	487	4.385	42	30	48
	R. Lingual gyrus	100	3.695	26	−92	−20
R. IOG seed	**HW > OB**					
	R. Middle occipital gyrus	2,553	−6.255	38	−72	0
	L. Middle occipital gyrus	1943	−5.251	−38	−82	0

The significance threshold was set at voxel-level *p* < 0.001 and cluster-level p_FDR_ < 0.05 for FC. FC, functional connectivity; MNI, Montreal Neurological Institute; FDR, false discovery rate; DLPFC, dorsolateral prefrontal cortex; INS, insula; IOG, inferior occipital gyrus; OB, obese individuals; HW, healthy weight individuals; L, left; R, right.

### 3.5 Relationships between neuronal and neurotransmitter systems

**Figure 4:**
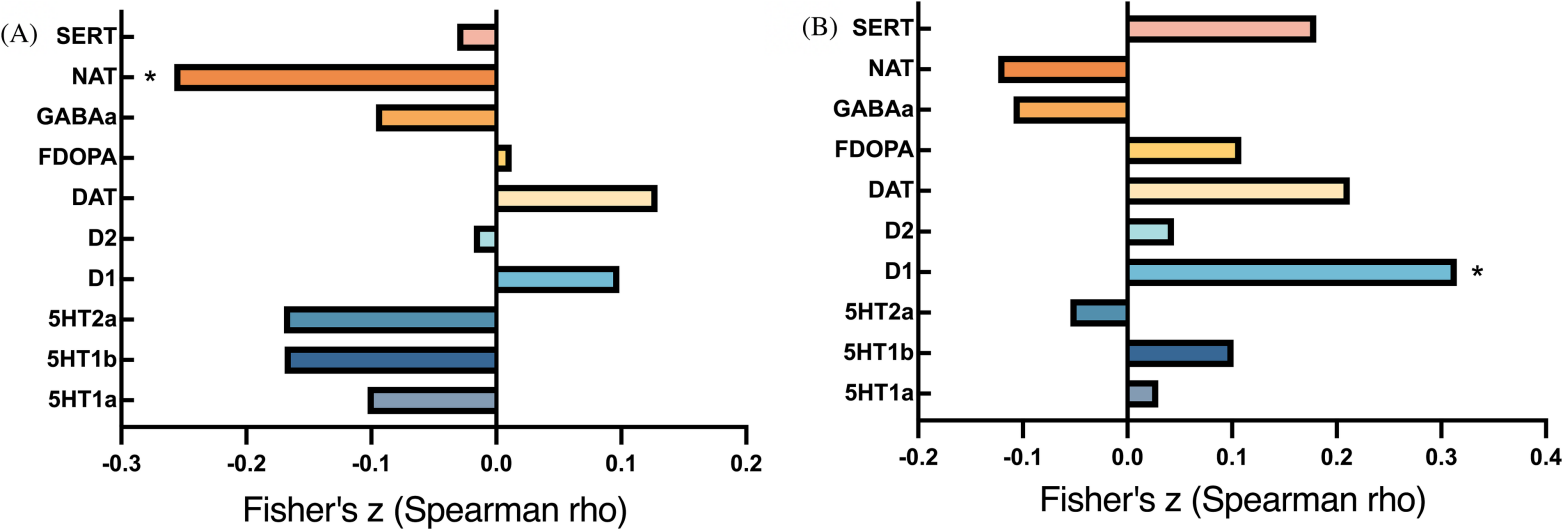
Bar plots of cross-modal correlations between receptor systems and ICC/fALFF components. **(A)** Correlations between ICC and receptor systems; **(B)** Correlations between fALFF and receptor systems. *p* < 0.05, false discovery rate corrected.

ICC changes between the two groups were significantly correlated with NAT (exact *p* = 0.004, P_FDR_ = 0.039) (Figure 4). In addition, changes in fALFF were spatially correlated with dopamine receptor (D1) (exact *p* < 0.001, P_FDR_ = 0.008) (Figure 4).

### 3.6 Mediation analysis results

For obese individuals, rDLPFC-PCL FC partially mediated the relationship between BMI and the DCCS scores. Additionally, fALFF values for the left insula, bilateral cerebellum crus 2, and right temporal pole each partially mediated the relationship between BMI and the area under curve (AUC) results for the DDT. For healthy weight individuals, no significant results were found in Mediation analyses. Detailed mediation analysis results are presented in Supplementary materials.

## 4 Discussion

In the current study, we examined the alterations of intrinsic functional architecture in obese group versus non-obese groups using a data-driven multiple-algorithm analysis combining ICC and fALFF with seed-based RSFC. Obese individuals, compared to healthy weight individuals, showed significant differences in the right DLPFC and left insula both in the intrinsic spontaneous activity and global connectivity index (i.e., fALFF and ICC). Seed-based FC analysis showed enhanced connectivity between the right DLPFC and the left precuneus, accompanied by reduced connectivity between the DLPFC and the sensorimotor cortex in the obese group versus healthy weight group. Additionally, using a seed placed in the left insula, the obese group exhibited significantly higher FC with the left IPL extending to the AG, right DLPFC, and lingual gyrus. Crucially, changes in ICC and fALFF correlated, respectively, with NAT and dopaminergic (D1) system. Mediation analyses revealed that fALFF in the left insula and the DLPFC-PCL FC partially mediated relations between BMI and impulsivity as well as cognitive flexibility. Taken together, these findings provide new neuroimaging evidence for possible neurophysiological mechanisms underlying obesity.

### 4.1 Alterations of the prefrontal control cortex and insula

In the current study, obese individuals exhibited increased spontaneous fluctuations in the right DLPFC. The DLPFC, in conjunction with the executive control network (ECN), has been implicated in the top-down control of appetitive processes and self-regulation of overeating (56, 57). Supporting this finding, recent studies have also demonstrated elevated spontaneous fluctuations in the DLPFC among overweight individuals (20, 118). Concurrently, structural imaging studies have observed an association between obesity and smaller DLPFC volumes (58) that are also negatively correlated with disinhibited eating (59). Dovetailing with spontaneous fluctuation data, obese individuals also had elevated global connectivity (i.e., ICC) in the right DLPFC which is posited to play a pivotal area in regulating obesogenic eating behaviors (60). Specifically, recent work has demonstrated that the suppression of food cravings in response to palatable foods activates the DLPFC (61), with the degree of activation correlating with subsequent weight loss success in a dietary interventions program (62–64). In addition, the neuromodulation and neurofeedback training targeting the DLPFC can reduce food craving and promote top-down regulation of appetite in obese individuals (65, 66).

In the subsequent FC analysis, the obese group showed comparatively lower FC between the right DLPFC and the sensorimotor network (i.e., left postcentral gyrus, left precentral gyrus, and bilateral PCL), which is regarded as a central hub and “engine of desire” connecting the body and brain in the neural vulnerability model of overeating (67). Importantly, we also found right DLPFC-PCL FC mediated a negative relationship between BMI and cognitive flexibility performance. Although obese participants may have attempted weight control, they also hold positive implicit attitudes and automatic reward tendencies toward palatable foods (68, 69). As such, these individuals may experience stronger conflicts between weight control goals and hedonic eating impulses than non-obese peers do (70). In line with these perspectives, obese people may expend more cognitive effort and resources to suppress their impulses as reflected by the increment of spontaneous activity in DLPFC (12).

Obese participants also showed aberrant alterations in the left insula both in spontaneous fluctuations and global connectivity. The insula is involved in the integrative processing of food craving, gustatory perception, and hedonic consumption (71–73) and has been identified as a high expression region of the obesity-susceptibility genes (74). In addition, as an important node of the salience network (SN), the insula also plays a crucial part in interoceptive awareness, conscious urges to food-seeking and the homeostasis regulation of hunger and satiety (75, 76). Consistent with our results, obese and overweight people had increased low-frequency fluctuation in the insula (20, 77). These findings also converge with evidence that obese individuals display higher activation of the insula in the reward processing (119). Prior research has also linked higher BMI with an augmented activation in the insula and frontal operculum during attention reallocation to appetizing food images (73). Moreover, under a pleasure mindset, obese individuals have been found to select larger food portions than the healthy weight individuals do, as reflected by heightened activation of the frontal operculum (120). Consequently, these findings suggest obese individuals more readily shift attention toward appetizing food cues and show augmented hedonic enjoyment, characterized by heightened spontaneous fluctuation and global connectivity in the insula and frontal operculum.

### 4.2 Aberrational spontaneous activity of brain regions in DMN

We also found that obese individuals have a higher fALFF value in the left mPFC and right IPL, known as important hubs of the DMN, implicated in self-reflection, decision-making, information integration, and episodic memory retrieval (78–80). Atypical activation of mPFC implicated in food anticipation, prediction error, and intertemporal choice has been observed in obesity and binge-eating disorder (81–84). A recent meta-analysis also reported consistently lower gray matter volume in the mPFC in obese samples (85). In addition, IPL engagement in priority coding, attentional shifting, and body-image representation (86) has been found to play a role in the alterations of spontaneous fluctuation and functional interaction of obese and binge eating disorder samples (87, 88). Moreover, obese individuals show greater default mode circuit activity under food cue-reactivity and resting state conditions (24, 89, 90). On the other hand, previous studies have demonstrated that weight loss interventions are associated with a reduction in intrinsic activity and local function synchrony in DMN (91, 92) that are accompanied by greater fat mass loss (92). Our findings of a stronger functional connection between the DMN and SN in obese individuals align with those of Lee et al. (93) who found enhanced RSFC between the DMN and SN is associated with body image distortions in individuals with eating disorders. Together, aberrations of intrinsic functional patterns in default mode circuits observed in obese individuals may reflect deficits in forming a healthy and comprehensive self-representation and ability in making sensible decisions, which would contribute to the loss of control during eating and abandonment of longer-term goals related to weight control in daily life (94, 95).

### 4.3 Aberrational FC based on the DLPFC and insula

Notably, FC analyses showed enhanced FC between (1) right DLPFC and left precuneus; (2) left insula and DMN (IPL/AG) in obese individuals; Moreover, we found enhanced FC between SN (insula) and ECN (DLPFC) in the obese group. The triple network model proposes that disrupted organization and functioning of the ECN, SN and DMN might be prominent features of various psychiatric and addictive disorders (96, 97). Deficits in functional interactions between these networks have been demonstrated in addiction and disordered eating based on resting-state imaging (97–99). Our results complement those of Boehm et al. (100) who found increased functional coupling between the SN and DMN among individuals with eating disorders. Functional interactions between the SN and DMN are also associated with self-control, which might modulate overeating and hedonic consumption of palatable food (101). In addition, a recent electroencephalographic (EEG) study observed increased delta connectivity between SN and ECN in problematic cannabis users that was accompanied by distributed patterns of excessive cannabis usage (102). According to the triple-network model, maladaptive patterns of dysfunctional connectivity in the ECN, SN, and DMN may manifest as disturbances in the capacity to integrate information from internally focused processing and externally focused processing, resulting in out of control eating and hedonic overconsumption among obese individuals.

### 4.4 Altered spontaneous activity of the cerebellum

There is increasing evidence that the cerebellum is involved in various higher-order cognitive processes (103), including impulse control (104), reward anticipation, and decision-making. The dysregulation of these functions is closely associated with addictive behaviors. Recent rsfMRI studies have demonstrated decreased intrinsic activity (e.g., ALFF and ReHo) in the cerebellum among obese individuals and smokers (105, 106), and stronger cravings (107). Zhu et al. (108) has proposed that activated cerebellar regions play a key role in integrating sensory, visceral, and affective signals related to appetite, taste, and olfaction during feeding or feeding control. This aligns with our findings that obese individuals exhibit decreased fALFF in the bilateral cerebellum compared to non-obese controls, suggesting a potential disruption in these processes in obesity. Impulsivity, as a risk factor for obesity, is associated with disinhibited eating (109) and atypical BOLD activation of the cerebellum for food odors among impulsive children compared to controls (110). A recent meta-analysis has also highlighted the cerebellum’s role in appetite control and behavioral regulation, with structural abnormalities observed in obesity (111). In line with these findings, we identified cerebellum crus2 fALFF as a partial mediator of the relationship between BMI and impulsivity. Overall, our results highlighting possible cerebellum involvement in impulsive control deficits and reward processing among obese individuals provide foundations for further related investigations.

### 4.5 Association between the brain and neurotransmitter systems

The disturbance of intrinsic functional architecture in obesity was also associated with noradrenaline and dopaminergic (D1) neurotransmitter systems activity. These pathways play key roles in the onset of impulsivity and overeating for obesity and metabolic syndrome (112). In an animal study, D1 receptors gene expression were associated with weight gain in overeating and proneness-to-obesity (113). In addition, prior research has shown that noradrenaline availability is related to subjective feelings of hunger in humans (38) while baseline noradrenaline levels predict the efficacy of subsequent weight loss (114). In obesity treatment, noradrenaline reuptake inhibitors have emerged as targets for anti-obesity interventions (115). Notably, our findings suggest that the distinct associations of ICC and fALFF with neurotransmitter systems may stem from their representation of different aspects of intrinsic brain function. In line with previous findings, the current study suggested that obesity-related dysfunctions may be associated with abnormalities in the dopaminergic and noradrenaline systems (116, 117).

### 4.6 Strengths and limitations of the research

The main strengths of this study included its relatively large sample size compared to numerous related studies, a methodological approach that facilitated the assessment of not only regional aberrations in spontaneous neural fluctuations but also FC and neurotransmitter involvement in obesity, and evaluation of neural influences that may partially explain why higher BMI levels are associated with behavioral performance deficits related to impulsivity as well as cognitive flexibility. Strengths aside, findings should be interpreted in light of the following limitations: First, because this study was cross-sectional, future research is needed to track dynamic changes that correspond with BMI alteration. Second, because sample was relatively young (ages 22–36), extensions are needed in samples with a broader age range as well as those within particular developmental stages (e.g., adolescents, older adults). Third, associations between spontaneous brain activity alterations and neurotransmitter receptor activity should be regarded as indirect evidence. Future research should employ simultaneous PET and MRI probes to obtain more direct evidence. Finally, because we focused on resting-state fMRI. Future research should also consider structural bases of functional abnormalities in obese individuals.

## 5 Conclusion

In summary, through ICC, fALFF, and FC analyses, the current study investigated links between obesity and intrinsic functional alterations using resting-state fMRI. Compared to non-obese peers, obese individuals showed dysfunctional spontaneous activity in the prefrontal cortex, insula, sensorimotor cortex, and default mode circuits. In addition, we observed functional interaction disturbances between key hubs in the three-network-model including the SN, ECN, and DMN among obese individuals. Finally, aberrations of intrinsic functional architecture were related to dopaminergic and noradrenaline neurotransmitter systems. The integration of neuroimaging and molecular perspectives might help characterize the neurophysiological mechanisms underlying obesity, potentially facilitating the development of more effective clinical interventions that could decrease its prevalence.

## Data Availability

Publicly available datasets were analyzed in this study. This data can be found here: the Human Connectome Project (http://www.humanconnectome.org/).

## Footnotes

^1^ https://www.who.int/zh/news-room/fact-sheets/detail/obesity-and-overweight

^2^ https://www.humanconnectome.org/study/hcp-young-adult

^3^ https://github.com/juryxy/JuSpace

^4^ http://processmacro.org/index.html
