# Supplementary material for: Obesity-related alterations of intrinsic functional architecture: a resting-state fMRI study based on the human connectome project
Source: Front Nutr. 2025 May 29;12:1559325. doi: 10.3389/fnut.2025.1559325 (PMC12158695; doi:10.3389/fnut.2025.1559325)
Supplement: Supplementary file 1 [file Data_Sheet_1.docx]

Supplementary Material

# Supplementary method and results

## The PET and SPECT map selections

The PET and SPECT maps included serotonin system (5HT1a, 5HT1b, 5HT2a and SERT) (Savli et al., 2012), dopaminergic system (D1, D2, DAT and FDOPA) (Alakurtti et al., 2015; Dukart et al., 2018; García-Gómez et al., 2013; Kaller et al., 2017), GABAergic receptor (García-Gómez et al., 2013) and NAT (Hesse et al., 2017).

## The mediation analysis results

Because BMI exhibited a significant negative association with the scores of DCCS, r = -0.14, P = 0.002) and DDT (r = -0.173, P < 0.001), we employed mediation analysis to elucidate the brain's role in these relationships. For the obesity group, the results indicated that the rDLPFC-PCL FC partially mediated the relationship between BMI and DCCS scores (indirect effect estimate = -0.0226, 95% CI = [-0.0507, -0.0015]; Fig. S1). Additionally, the fALFF of the left insula (indirect effect estimate = 0.0294, 95% CI = [0.0074, 0.0553]; Fig. S2), left cerebellum crus 2 (indirect effect estimate = -0.0363, 95% CI = [-0.0629, -0.0125]; Fig. S2), right cerebellum crus 2 (indirect effect estimate = -0.0306, 95% CI = [-0.0536, -0.0120]; Fig. S2), and right temporal pole (indirect effect estimate = -0.0267, 95% CI = [-0.492, -0.0070]; Fig. S2) each partially mediated the relationship between BMI and the AUC of DDT. We have conducted a mediation analysis for the healthy weight group, and the results showed no significant findings. Specifically, the mediation effect of rDLPFC-PCL FC on the relationship between BMI and DCCS scores was not significant (indirect effect estimate = 0.0001, 95% CI = [-0.0074, 0.0079]). Similarly, the mediation effects of the fALFF of the left insula (indirect effect estimate = 0.0033, 95% CI = [-0.0131, 0.0211]), left cerebellum crus 2 (indirect effect estimate = 0.0025, 95% CI = [-0.0089, 0.0184]), right cerebellum crus 2 (indirect effect estimate = 0.0066, 95% CI = [-0.0095, 0.0269]), and right temporal pole (indirect effect estimate = -0.0029, 95% CI = [-0.0203, 0.0143]) on the relationship between BMI and the AUC of DDT were also not significant.

## Reference

Alakurtti, K., Johansson, J.J., Joutsa, J., Laine, M., Bäckman, L., Nyberg, L., Rinne, J.O., 2015. Long-term test-retest reliability of striatal and extrastriatal dopamine D2/3 receptor binding: study with [(11)C]raclopride and high-resolution PET. J Cereb Blood Flow Metab, 35, 1199–1205. https://doi.org/10.1038/jcbfm.2015.53

Dukart, J., Holiga, Š., Chatham, C., Hawkins, P., Forsyth, A., McMillan, R., Myers, J., Lingford-Hughes, A.R., Nutt, D.J., Merlo-Pich, E., Risterucci, C., Boak, L., Umbricht, D., Schobel, S., Liu, T., Mehta, M.A., Zelaya, F.O., Williams, S.C., Brown, G., Paulus, M., Honey, G.D., Muthukumaraswamy, S., Hipp, J., Bertolino, A., Sambataro, F., 2018. Cerebral blood flow predicts differential neurotransmitter activity. Sci Rep-uk, 8, 4074. https://doi.org/10.1038/s41598-018-22444-0

García-Gómez, F.J., García-Solís, D., Luis-Simón, F.J., Marín-Oyaga, V.A., Carrillo, F., Mir, P., Vázquez-Albertino, R.J., 2013. [Elaboration of the SPM template for the standardization of SPECT images with 123I-Ioflupane]. Rev Esp Med Nucl Imagen Mol, 32, 350–356. https://doi.org/10.1016/j.remn.2013.02.009

Hesse, S., Becker, G.-A., Rullmann, M., Bresch, A., Luthardt, J., Hankir, M.K., Zientek, F., Reißig, G., Patt, M., Arelin, K., Lobsien, D., Müller, U., Baldofski, S., Meyer, P.M., Blüher, M., Fasshauer, M., Fenske, W.K., Stumvoll, M., Hilbert, A., Ding, Y.-S., Sabri, O., 2017. Central noradrenaline transporter availability in highly obese, non-depressed individuals. Eur J Nucl Med Mol I, 44, 1056–1064. https://doi.org/10.1007/s00259-016-3590-3

Kaller, S., Rullmann, M., Patt, M., Becker, G.-A., Luthardt, J., Girbardt, J., Meyer, P.M., Werner, P., Barthel, H., Bresch, A., Fritz, T.H., Hesse, S., Sabri, O., 2017. Test-retest measurements of dopamine D1-type receptors using simultaneous PET/MRI imaging. Eur J Nucl Med Mol I, 44, 1025–1032. https://doi.org/10.1007/s00259-017-3645-0

Savli, M., Bauer, A., Mitterhauser, M., Ding, Y.-S., Hahn, A., Kroll, T., Neumeister, A., Haeusler, D., Ungersboeck, J., Henry, S., Isfahani, S.A., Rattay, F., Wadsak, W., Kasper, S., Lanzenberger, R., 2012. Normative database of the serotonergic system in healthy subjects using multi-tracer PET. Neuroimage, 63, 447–459. https://doi.org/10.1016/j.neuroimage.2012.07.001

# Supplementary Figures and Tables

## Supplementary Figures

### Figure S1.


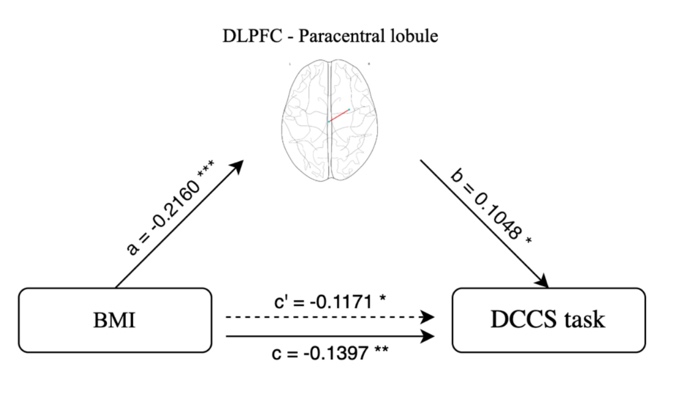


**Supplementary Figure S1.** The mediated model. The mediation analysis indicated that the functional connectivity between DLPFC and paracentral lobule could partially mediate the relationship between BMI and the scores of DCCS. Notes: * p < 0.05, ** p < 0.01, *** p < 0.001. DLPFC, dorsolateral prefrontal cortex; BMI, body mass index; DCCS, Dimensional Change Card Sort Task.

### Figure S2.


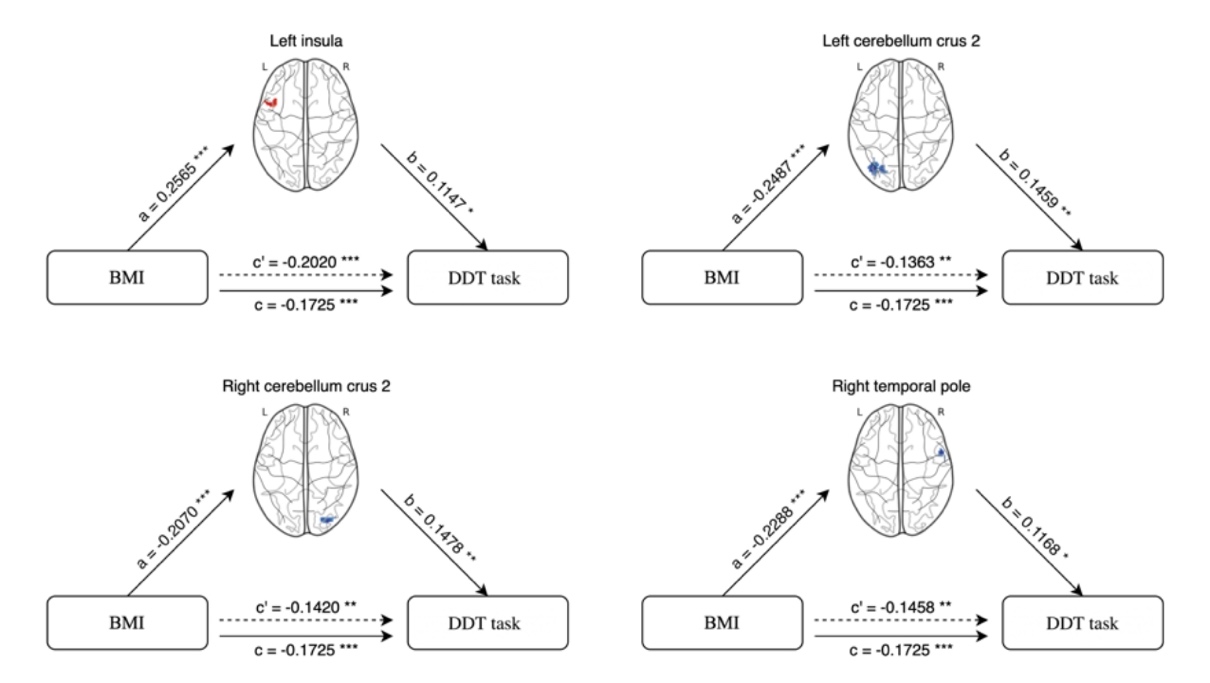


**Supplementary Figure S2.** The mediation analysis indicated that the fALFF of the left insula, bilateral cerebellum crus 2, and right temporal pole could partially mediate the relationship between BMI and the AUC of DDT. Notes: * p < 0.05, ** p < 0.01, *** p < 0.001. BMI, body mass index; AUC, area under curve; DDT, Delay Discounting Task.

## Supplementary Table

### Table S1.

**Supplementary Table S1.** D**emographic and Clinical Characteristics of the Participants**

|  | Obese people | | healthy-weight people | | T/χ² | P-value |
| --- | --- | --- | --- | --- | --- | --- |
| Total N | 198 | | 291 | |  |  |
| **Continuous variables** | Mean | SD | Mean | SD |  |  |
| Age (years) | 29.25 | 3.71 | 28.67 | 3.79 | 1.677 | 0.094 |
| Handedness | 66.94 | 43.07 | 68.01 | 41.49 | -0.274 | 0.784 |
| BMI (kg/m²) | 36.17 | 3.45 | 22.09 | 1.23 | 47.618 | <0.001 |
| CardSort_AgeAdj | 100.51 | 10.63 | 102.73 | 9.17 | -2.461 | 0.014 |
| Flanker_AgeAdj | 101.70 | 9.87 | 101.64 | 10.18 | 0.06 | 0.952 |
| DDisc_AUC_mean | 0.34 | 0.21 | 0.40 | 0.22 | -3.12 | 0.002 |
| **Categorical variables** | N | % | N | % |  |  |
| Gender |  |  |  |  | 0.688 | 0.407 |
| Male | 85 | 42.7 | 114 | 57.3 |  |  |
| Female | 113 | 39.0 | 177 | 61.0 |  |  |
| Ethnicity |  |  |  |  | 0.923 | 0.337 |
| Hispanic | 20 | 47.6 | 22 | 52.4 |  |  |
| Non-Hispanic | 178 | 40.0 | 267 | 60.0 |  |  |

**Abbreviation**: BMI, body mass index (kg/m2); SD, standard deviation; CardSort_AgeAdj, Age-adjusted scores of Dimensional Change Card Sort Test; Flanker_AgeAdj, Age-adjusted scores of Flanker Task; DDisc_AUC_mean the average values of are under the curve (AUC) for discounting of the $200 and $40,000.
